# Deletion of *Gpatch2* does not alter *Tnf* expression in mice

**DOI:** 10.1038/s41419-023-05751-x

**Published:** 2023-03-27

**Authors:** Destiny Dalseno, Holly Anderton, Andrew Kueh, Marco J Herold, John Silke, Andreas Strasser, Philippe Bouillet

**Affiliations:** 1 The Walter and Eliza Hall Institute of Medical Research, Parkville, VIC 3052 Australia; 2 Department of Medical Biology, The University of Melbourne, Melbourne, VIC 3052 Australia

**Keywords:** Mechanisms of disease, Inflammation

## Abstract

The cytokine TNF has essential roles in immune defence against diverse pathogens and, when its expression is deregulated, it can drive severe inflammatory disease. The control of TNF levels is therefore critical for normal functioning of the immune system and health. We have identified GPATCH2 as a putative repressor of *Tnf* expression acting post-transcriptionally through the *TNF* 3’ UTR in a CRISPR screen for novel regulators of TNF. GPATCH2 is a proposed cancer-testis antigen with roles reported in proliferation in cell lines. However, its role in vivo has not been established. We have generated *Gpatch2*^*−/−*^ mice on a C57BL/6 background to assess the potential of GPATCH2 as a regulator of *Tnf* expression. Here we provide the first insights into *Gpatch2*^*−/−*^ animals and show that loss of GPATCH2 affects neither basal *Tnf* expression in mice, nor *Tnf* expression in intraperitoneal LPS and subcutaneous SMAC-mimetic injection models of inflammation. We detected GPATCH2 protein in mouse testis and at lower levels in several other tissues, however, the morphology of the testis and these other tissues appears normal in *Gpatch2*^*−/−*^ animals. *Gpatch2*^*−/−*^ mice are viable, appear grossly normal, and we did not detect notable aberrations in lymphoid tissues or blood cell composition. Collectively, our results suggest no discernible role of GPATCH2 in *Tnf* expression, and the absence of an overt phenotype in *Gpatch2*^*−/−*^ mice warrants further investigation of the role of GPATCH2.

## Introduction

Tumour necrosis factor (TNF) is a major pro-inflammatory cytokine with diverse cellular functions in immune defence against pathogens. Defects in the control of *TNF* expression are associated with several human pathologies, including rheumatoid arthritis and inflammatory bowel disease [1]. Post-transcriptional regulation of *TNF* is essential for maintaining appropriate TNF levels in tissues and this relies upon discrete regulatory sequences within the *TNF* 3’ untranslated region (UTR) which in interactions with RNA-binding proteins (RBPs) regulate *TNF* mRNA stability, translation and splicing [2, 3]. *TNF 3*’ UTR regulatory elements include the AU-rich element (ARE) and constitutive decay element (CDE) which mediate *TNF* mRNA decay upon binding by RBPs [4, 5]. We have previously identified and characterised the *TNF* New Regulatory Element (NRE) [6], and showed that heterozygosity for concomitant deletion of the ARE & NRE leads to embryonic death due to excess TNF [7]. We also showed that specific deletion of the ARE gives rise to inflammation of the digestive tract and mild arthritis, while combined deletion of the ARE & CDE yielded vascular degeneration and embryonic lethality around E15. Given the clear necessity of the *TNF* 3’ UTR in maintaining appropriately low levels of TNF in vivo, we sought to identify novel regulators of *TNF* mRNA that act through the *TNF* 3’ UTR. GPATCH2 was identified as a putative repressor of *Tnf* expression in a CRISPR *Tnf* reporter screen. This protein belongs to the G-patch domain-containing family of proteins. Members of the G-patch family are varied and diverse in function. However, the G-patch domain, characterised by six highly conserved glycine residues, is present in a number of proteins associated with RNA processing and splicing and is as such a predicted RNA-binding domain [8].

GPATCH2 is a proposed cancer-testis antigen, as expression of *Gpatch2* mRNA is high in rat and human testis tissue [9, 10] while elevated *GPATCH2* mRNA has been noted in human breast cancer samples [10]. GPATCH2 has been implicated in cell proliferation in vitro [9, 10], however, the role of GPATCH2 in vivo has not been established. We generated *Gpatch2*^*−/−*^ mice to investigate the role of GPATCH2 in *Tnf* expression in vivo. Here we characterise *Gpatch2*^*−/−*^ animals and show that loss of GPATCH2 does not affect basal TNF levels in mice and also does not alter *Tnf* expression in lipopolysaccharide or SMAC-mimetic injection models of inflammation.

## Results

### CRISPR screen identifies GPATCH2 as a putative repressor of *TNF* expression

**Fig. 1:**
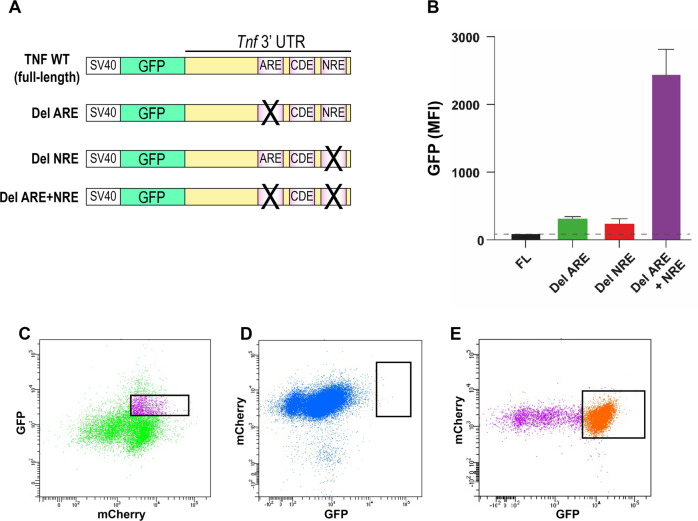
CRISPR screen for identification of novel *Tnf* regulators. **A** Schematic representation of the wild-type *Tnf* 3’ UTR and ARE or NRE deletion (Del) *Tnf* 3’ UTR GFP reporter constructs. The reporter constructs were comprised of the SV40 promoter sequence, GFP coding sequence and a mouse *Tnf* 3’ UTR sequence, such that *GFP* mRNA is regulated by the *Tnf* 3’ UTR. **B** Combined deletion of the ARE and NRE yields considerable stabilisation of a *GFP-Tnf* 3’ UTR reporter construct resulting in increased GFP signal. Constructs were expressed in HEK293T cells before flow cytometric analysis of GFP expression. GFP mean fluorescence intensity (geometric) was plotted. **C** Representative flow cytometry plot showing HEK293T cells expressing Cas9 (mCherry) and moderate *Tnf-GFP* reporter expression were selected for clonal expansion and subsequent transduction with the GeCKO sgRNA library. **D** Cells with very high GFP after transduction with the sgRNA library were selected for expansion. **E** Following expansion, cells with high GFP were selected for sequencing of the enriched CRISPR sgRNAs.

The *TNF* 3’UTR contains sequences that participate in the post-transcriptional regulation of this gene. Using a panel of reporter constructs missing one or two of these regulatory elements (Fig. 1A), we have previously shown that deletion of the AU-rich element (ARE) or the new regulatory element (NRE) increases the basal expression of the *Tnf* reporter gene, and that concomitant loss of both elements increased its expression to a very high level (Fig. 1B) [6]. These results have now been confirmed in vivo [7]. Since the ARE has been shown to be the target of several RNA-binding proteins, we hypothesised that the function of the NRE is similar and that several RBPs may cooperate in vivo to maintain low TNF levels and thereby avoid pathological inflammation. In order to identify potential RBPs involved in this process, we engineered a CRISPR screen that mimics the effect of the deletion of both negative regulatory processes for the control of *Tnf* expression by ablating the effect of the ARE when using the *Del NRE* construct and the effect of the NRE when using the *Del ARE* construct (Fig. 1A).

Cas9-mCherry-expressing HEK293T cells were stably transfected with *GFP-Tnf Del ARE* or *GFP-Tnf Del NRE* reporter constructs (Fig. 1A) and selected with hygromycin. Single mCherry-Cas9 expressing HEK293T cell clones with moderate GFP reporter expression were selected (Fig. 1C) and expanded, and six independent clones were infected with the lentiviral Human GeCKO v2 [11] sgRNA library and selected by adding puromycin. Seven days following infection, GFP-high cells were sorted (Fig. 1D) and then expanded in culture. After expansion, enriched GFP-high cells were sorted (Fig. 1E) and their DNA was immediately prepared for analysis of their sgRNA content. After expansion of high-GFP clones following infection of *GFP-Tnf Del ARE* expressing cells with the GeCKO library, GFP expression was lost, in contrast to *GFP-Tnf Del NRE* expressing cells which retained high GFP expression after infection and sorting. We therefore proceeded to sequence only the high-GFP clones harbouring the *GFP-Tnf Del NRE* construct (the results from this CRISPR screen are presented in Supplementary Table 1).

In this screen, increased GFP fluorescence following application of the CRISPR library indicated greater reporter mRNA stability, presumably through the loss of a negative regulator acting on the *Tnf* 3’ UTR. From our reporter screen utilising an NRE deletion construct, high *GPATCH2* sgRNA counts were present in two independent GFP-high clones. The presence of a unique *GPATCH2* sgRNA in each of these two clones, one targeting exon 3 of *GPATCH2* and the other exon 7, suggested a true effect on reporter expression due to specific loss of GPATCH2. Indeed, GPATCH2 was the only candidate that was found more than once in this screen, and since the G-patch domain is found in proteins involved in RNA processing, GPATCH2 was a particularly promising candidate for a novel regulator of *Tnf* expression. Other hits supported the relevance of this CRISPR screen, including HnRNPA0, a known *Tnf* 3’ UTR RNA-binding protein [12, 13], as well as several genes associated with autoimmune inflammatory disease, including AFF3 and TNIP1 [14, 15]. To evaluate whether GPATCH2 plays a role in regulation of *Tnf* expression at the level of the whole organism, we proceeded immediately to ablating the *Gpatch2* gene in the mouse, anticipating that loss of GPATCH2 would deregulate *Tnf* expression and cause an inflammatory phenotype.

### *Gpatch2*^*−/−*^ animals are healthy and develop normally

**Fig. 2:**
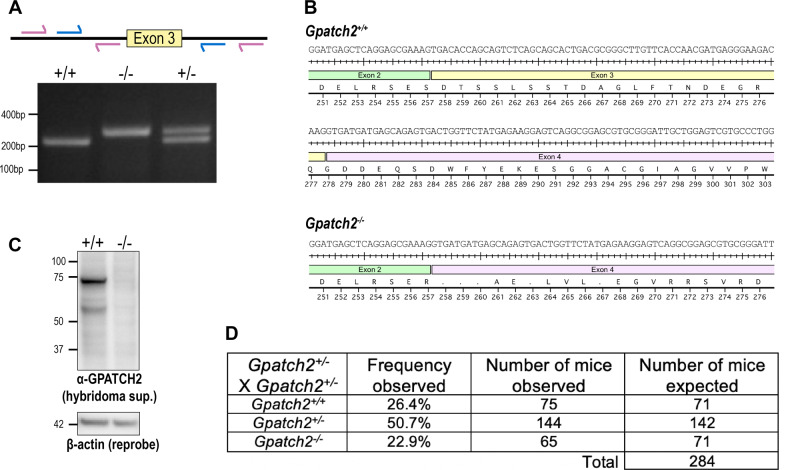
Generation and validation of *Gpatch2*^*−/−*^ mice. **A** *Gpatch2* primers and genotyping. Schematic representation of primers located in the introns flanking exon 3 of *Gpatch2* for CRISPR-targeted deletion of exon 3 (blue) and genotyping (purple) to distinguish wild-type and *Gpatch2* mutant alleles. **B** Exon 3 was deleted by CRISPR/Cas9 to cause a frameshift in *Gpatch2*^−/−^ mice. **C** Immunoblotting for GPATCH2 in lysates from the testis of wild-type and *Gpatch2*^*−/−*^ mice with hybridoma supernatant. **D** Observed numbers of wild-type, heterozygous *Gpatch2*^*+/−*^, and homozygous *Gpatch2*^*−/−*^ offspring obtained from inter-crosses of *Gpatch2*^*+/−*^ mice.

*Gpatch2*^*−/−*^ mice on a C57BL/6 background were generated by CRISPR/Cas9 technology [16]; sgRNA primers flanking exon 3 were used to excise exon 3 to knock out *Gpatch2* (Fig. 2A). Exon 3 was targeted to knock out GPATCH2 as its deletion results in a frame shift with several stop codons generated immediately after exon 2 (Fig. 2B). Founder mice with the expected deletion were identified by PCR and the deletion was confirmed by Sanger sequencing. Mutant founders were then crossed to wild-type C57BL/6 mice to generate true heterozygotes which were then inter-crossed to generate homozygous mutants. The different genotypes were distinguished with a 3-primer PCR (Fig. 2A).

Throughout a 2-year observation period, *Gpatch2*^*+/−*^ (*n* > 200) and *Gpatch2*^*−/−*^ (*n* > 200) mice developed and aged normally and mice of the three expected genotypes were obtained from inter-crosses of *Gpatch2*^*+/−*^ mice at the predicted Mendelian ratios (Fig. 2D). To validate successful CRISPR targeting and knockout of the *Gpatch2* gene, we tested several commercially available antibodies against GPATCH2, but unfortunately without successful detection of endogenous GPATCH2 (data not shown). Therefore, we generated a GPATCH2 specific monoclonal antibody by immunising rats with a N-terminally truncated recombinant mouse GPATCH2 protein comprised of exons 4–10. Lysates from diverse organs from wild-type and *Gpatch2*^*−/−*^ (negative control) mice were screened with hybridoma supernatants for GPATCH2 expression, and several were successful in detecting endogenous GPATCH2 protein in the testis (data not shown). With such antibodies we then confirmed successful loss of GPATCH2 expression in *Gpatch2*^*−/−*^ mice (Fig. 2C). We cannot exclude the possibility that a truncated N-terminal GPATCH2 protein may exist in *Gpatch2*^*−/−*^ animals, however, the G-patch domain encoded by exon 10, that we assume to be essential for the function of this protein, was successfully deleted.

Next, we screened lysates from several organs for GPATCH2 expression with our most sensitive hybridoma supernatant, with lysates prepared from tissues of a *Gpatch2*^*−/−*^ mouse serving as a negative control. GPATCH2 was readily detected in wild-type testis tissue, and at lower levels also in several other tissues, including the brain, intestine, spleen and thymus (Supplementary Fig. 1). Of note, in many tissues from wild-type but not *Gpatch2*^*−/−*^ mice, the antibody detected a protein around 50 kDa (Supplementary Fig. 1). This suggests that a ~50 kDa isoform of GPATCH2 is expressed alongside full-length GPATCH2 in mouse tissues.

**Fig. 3:**
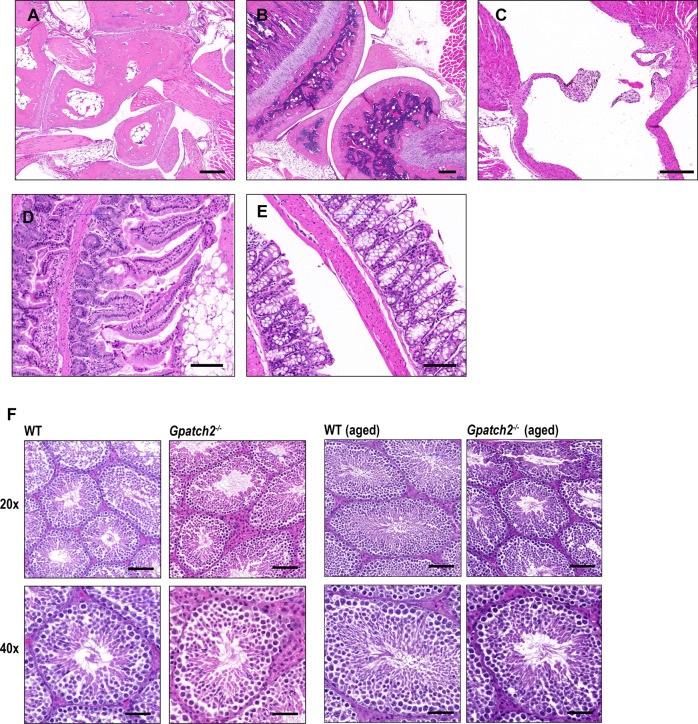
*Gpatch2*^*−/−*^ mice do not spontaneously develop inflammatory disease. Representative H&E staining of the ankle joint (**A**), knee joint (**B**), aortic root & valves (**C**) small intestine (**D**) and colon (**E**) in *Gpatch2*^*−/−*^ mice. **F** Representative images of testis morphology from wild-type (WT) and *Gpatch2*^*−/−*^ males at 8 weeks or 44 weeks (aged). Scale bars: **A**–**C** 200 μm, **D** and **E** 100 μm, **F** 20×: 100 μm, 40×: 50 μm.

**Fig. 4:**
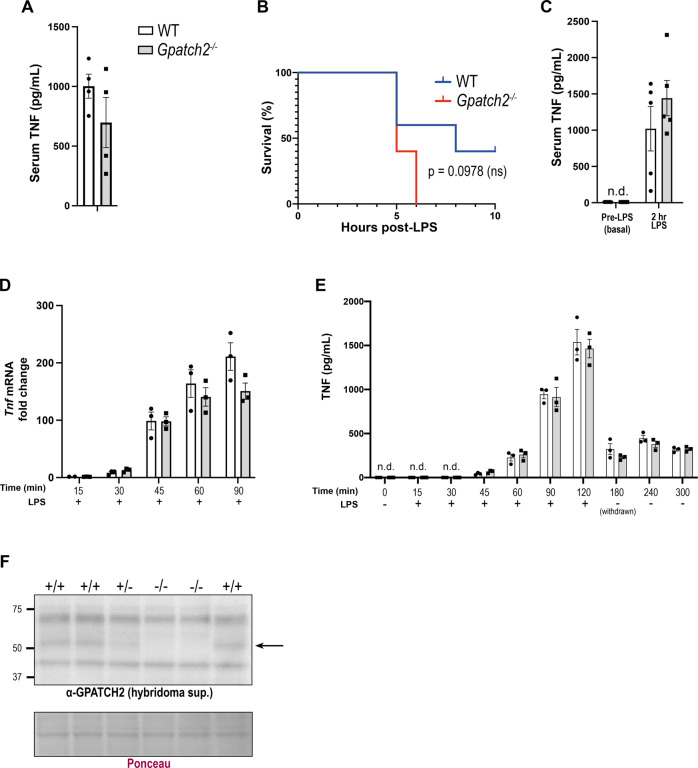
The absence of GPATCH2 does not affect LPS-induced *Tnf* expression in mice or BMDMs. **A** Mice were injected with 18 mg/kg LPS, and 3 h post-injection serum TNF levels were determined by ELISA (*n* = 4 mice per genotype). (*n* = 1) **B** Kaplan–Meier survival curves of wild-type and *Gpatch2*^*−/−*^ mice following injection with 5 mg/kg LPS (*n* = 5 mice per genotype). Uptick represents mice that did not reach ethical endpoint during the LPS challenge, and were sacrificed in a healthy state 10 h post-injection. (*n* = 1) **C** Serum TNF levels in mice of the indicated genotypes before and 2 h after injection of 5 mg/kg LPS. **D** Fold-change in *Tnf* mRNA plotted relative to *Hprt* mRNA in BMDMs of the indicated genotypes treated with LPS for the indicated times, as determined by qPCR (*n* = 3 mice per genotype). (*n* = 1) **E** Levels of TNF in the culture supernatant from BMDMs of the indicated genotypes following LPS treatment for the indicated times as determined by ELISA. LPS-supplemented culture medium was replaced 120 min post-LPS treatment and replaced with medium without LPS. **F** BMDMs of the indicated genotypes were treated with 100 ng/mL LPS for 2 h before lysis, and extracts were subjected to immunoblotting to detect GPATCH2 with concentrated hybridoma supernatant. GPATCH2 is indicated with an arrow, and lanes show BMDMs from individual mice (*n* = 3 wild-type, *n* = 1 *Gpatch2*^*+/−*^*,* *n* = 2 *Gpatch2*^*−/−*^*)*. (*n* = 1). White bars correspond to wild-type (WT) and grey bars to *Gpatch2*^*−/−*^ BMDMs throughout. Data are presented as mean ± SEM, all data points represent individual mice. n.d. not detected. ns not significant. No statistically significant *p* values were obtained from comparison of wild-type and *Gpatch2*^*−/−*^ mice (**A**, **C**–**E** Mann–Whitney test, **B** log-rank test).

Considering that serum TNF levels are undetectable in unchallenged *Gpatch2*^*−/−*^ mice (Fig. 4C, see pre-LPS serum TNF), it is not surprising that *Gpatch2* mutants did not display an increased propensity for TNF-related arthritis, inflammatory bowel disease or heart valve disease (Fig. 3A–E), pathologies that are observed in several of our *Tnf* 3’ UTR mutant mouse strains [6, 7]. Histological analysis of other tissues from *Gpatch2*^*−/−*^ mice also revealed no abnormalities (Supplementary Fig. 2). GPATCH2 was first described as a testis antigen and we confirmed expression of GPATCH2 in this tissue (Fig. 2C). However, testis tissue in both young and aged males appeared unaffected by the loss of GPATCH2 (Fig. 3F), and *Gpatch2*^*−/−*^ males were fertile (*Gpatch2*^*−/−*^ females were also fertile). To screen for alterations to the immune system, lymphoid organ cell numbers and cell subset composition were examined by flow cytometry in male and female *Gpatch2*^*−/−*^ mice at 6–14 weeks. No clear differences were observed in mutants compared to wild-type controls, with the exception of a statistically significant albeit small reduction in the frequency of B cells in the blood of *Gpatch2*^*−/−*^ male mice relative to age-matched control males (Supplementary Fig. 3). Other haematological parameters measured by automated blood analysis also yielded results comparable between *Gpatch2*^*−/−*^ and wild-type mice (Supplementary Fig. 4).

### Loss of GPATCH2 does not affect response to LPS

The roles of different *Tnf* 3’ UTR RNA-binding proteins are varied and context-dependent. For example, deletion of the ARE-binding protein TTP results in excess TNF-driven inflammation in vivo [17]. By contrast, the ARE-binding protein AUF1 exerts a protective effect following LPS administration in mice correlating with reduced *Tnf* and *IL-1β* mRNA levels, but deletion of AUF1 does not promote systemic inflammation [18]. Because of the absence of an overt phenotype in *Gpatch2*^*−/−*^ mice, and no detectable increase in baseline serum TNF levels in these animals (Fig. 4C), we next sought to evaluate whether loss of GPATCH2 may alter TNF responses in vivo. Intraperitoneal injection of lipopolysaccharide (LPS) induces a systemic inflammatory response in mice where binding of LPS to the TLR4/MD2 complex activates NF-κB transcription factors, leading to production of pro-inflammatory cytokines, including secretion of large quantities of TNF, from monocytic cells [19].

Wild-type and *Gpatch2*^*−/−*^ mice were injected with 18 mg/kg body weight LPS [20], and all mice showed a similar decrease in body temperature over 3 h post-injection regardless of genotype, and needed to be euthanised at this time due to reaching the ethically-determined endpoint of a 33 °C temperature. Blood serum collected post-mortem revealed comparable serum TNF levels between wild-type and *Gpatch2*^*−/−*^ mice (Fig. 4A). To slow down the development of symptoms, mice were next injected with 5 mg/kg LPS in order to detect potential differences in response between wild-type and *Gpatch2*^*−/−*^ mice. Mice were bled pre-LPS injection to establish baseline serum TNF levels, and TNF was undetectable in the serum from the *Gpatch2*^*−/−*^ animals (Fig. 4C). No difference in severity of pathology or serum TNF levels 2 h post-injection (Fig. 4B, C) were observed at this lower dose of LPS between wild-type and *Gpatch2*^*−/−*^ mice.

Since macrophages are the main producers of TNF, we also stimulated bone marrow-derived macrophages (BMDMs) from wild-type and *Gpatch2*^*−/−*^ mice with LPS in vitro. Induction of *Tnf* expression was assessed by qPCR, and *Tnf* mRNA levels were found to be comparable between wild-type and *Gpatch2*^*−/−*^ BMDMs following LPS treatment (Fig. 4D). TNF levels in culture supernatants remained similar between wild-type and *Gpatch2*^*−/−*^ BMDMs throughout LPS treatment, and also after withdrawal of LPS (Fig. 4E). GPATCH2 protein expression was confirmed to be present in non-stimulated wild-type BMDMs (data not shown), as well as in LPS-treated wild-type BMDMs but, as expected, absent in *Gpatch2*^*−/−*^ BMDMs (Fig. 4F). Despite expression of GPATCH2 in BMDMs, our in vivo and in vitro analysis of LPS-induced *Tnf* expression suggested no discernible role for GPATCH2 in the control of *Tnf* expression in these LPS stimulation models.

### GPATCH2 does not affect *Tnf* expression in the SMAC-mimetic-induced skin inflammation model

To further evaluate a potential role for GPATCH2 in the control of *Tnf* expression, we next examined the impact of loss of GPATCH2 in the context of TNF-dependent skin inflammation. SMAC-mimetic compounds (SMs) antagonise inhibitor of apoptosis proteins (IAPs), predisposing cells to cell death. Subcutaneous injection of the SMAC-mimetic Compound A (CompA) [21] depletes IAPs in the skin surrounding the site of injection, and a lesion is formed as a result of a localised acute inflammatory reaction within the epidermis [22]. This inflammatory reaction in the skin is TNF-dependent, as *Tnfr1*^*−/−*^ mice do not develop such a reaction following CompA injection [22].

**Fig. 5:**
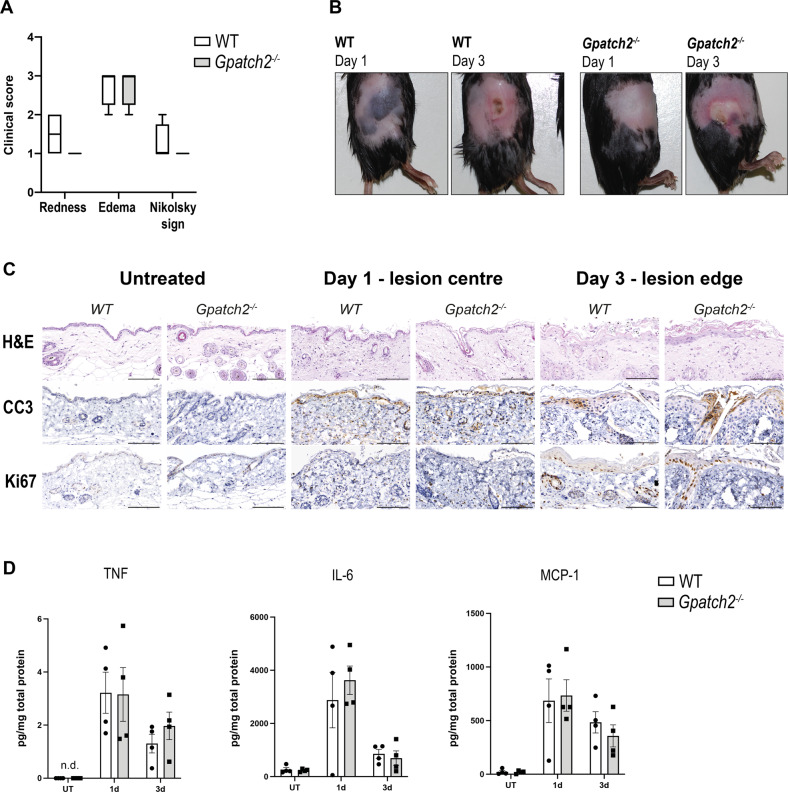
Loss of GPATCH2 does not alter the response to subcutaneous injection of a SMAC-mimetic. **A** Clinical scoring of SM-induced lesions in wild-type and *Gpatch2*^−/−^ mice. Box plot whiskers show minimum and maximum scoring values. **B** Representative images of SM-induced lesions in wild-type or *Gpatch2*^−/−^ mice 1 day or 3 days post-injection. **C** Representative images of SM-induced skin lesion centres in wild-type or *Gpatch2*^−/−^ mice stained with H&E, for CC3 or Ki67. Scale bars: 50 μm. **D** Cytokine levels in lysates from skin lesions in untreated, day 1 or day 3 mice of the indicated genotypes were determined by ELISA, and cytokine levels are shown in pg per mg of total protein in skin lysates. White bars correspond to wild-type (WT) and grey bars to *Gpatch2*^*−/−*^ mice throughout. Data are presented as mean ± SEM, data points represent individual mice (*n* = 4 per genotype). n.d. not detected. No statistically significant *p* values were obtained from comparison of wild-type and *Gpatch2*^*−/−*^ mice (Mann–Whitney test). (*n* = 1).

Mice were injected subcutaneously with CompA on one side of the flank, and injected with CompA on the opposite flank two days later for the 1 and 3 day timepoints. Unaffected skin was collected from the ventral thoracic region of each mouse as untreated control skin. Mice were euthanised three days after the first CompA injection, and hair removed with a depilatory cream before photography of lesions, as previously described [22]. Day 3 lesions were scored for redness and oedema, as well as the Nikolsky sign, an indicator of epidermal detachment [23]. No clear macroscopic differences were observed between the inflammatory lesions of wild-type and *Gpatch2*^*−/−*^ animals (Fig. 5A, B). H&E staining of lesions revealed comparable epidermal and dermal injury between wild-type and *Gpatch2*^*−/−*^ mice, and staining for cleaved (i.e. activated) caspase-3 (CC3) revealed no difference in the quantity or pattern of apoptotic cell death at both 1 day or 3 days post SM-injection (Fig. 5C). Staining for the nuclear protein Ki67 demonstrated comparable cell proliferation in wild-type and *Gpatch2*^*−/−*^ lesions (Fig. 5C). Day 1 lesion centres and day 3 lesion edges are shown in Fig. 5, as responses to CompA injection are most apparent at the site of injection, or lesion centre, on day 1 where considerable keratinocyte death occurs, and at the lesion edges on day 3 where wound healing is occurring. The absence of GPATCH2 also had no apparent impact on the expression of the inflammatory cytokines TNF, IL-6 and MCP-1 in skin lesion lysates at either 1 day or 3 days post-injection of SM (Fig. 5D). Collectively, these results show that *Gpatch2*^*−/−*^ mice do not display a heightened response to SM injection, nor elevated TNF in SM-induced lesions. This indicates that GPATCH2 is not essential to regulate *Tnf* expression in the SM injection model of inflammatory skin disease.

## Discussion

In a CRISPR screen using a *Tnf* reporter system, we identified GPATCH2 as a putative repressor of *Tnf* expression acting through the *Tnf* 3’ UTR. The presence of two unique *GPATCH2* targeting sgRNAs in our CRISPR screen was indicative of a true hit, which we aimed to confirm though ablation of *Gpatch2* in a mouse model. However, our present data collectively suggest that GPATCH2 is not critical for the control of *Tnf* expression in mice. Although loss of GPATCH2 caused increased *GFP-Tnf Del NRE* reporter expression in HEK293T cells in our CRISPR screen, this finding did not translate to a defect in the control of *Tnf* expression within the whole organism. Several reasons may be invoked to explain this result. In examining *Tnf* reporter expression, our CRISPR screen did not distinguish between proteins directly bound to the *Tnf* 3’ UTR, and those which altered *Tnf* expression indirectly by regulating other mRNA metabolism processes. GPATCH2 is reported to localise within nuclear speckles [10]. Nuclear speckles are enriched in splicing factors, and act as sites for several RNA processing steps [24]. In humans and mice, the G-patch protein family is comprised of approximately 25 members, many of which are involved in mRNA processing [25]. Several G-patch proteins, including GPATCH2, are reported to interact with the splicing factor DHX15 [10, 25]. While we did not investigate whether GPATCH2 has a role in *Tnf* mRNA splicing, it remains possible that GPATCH2 was identified in our screen due to a splicing-related role. Functional overlap with one or more other members of the G-patch family may therefore account for our observation of normal *Tnf* expression in *Gpatch2*^*−/−*^ mice, as well as the absence of an overt phenotype in *Gpatch2*^*−/−*^ mice.

Redundance in function between different RNA-binding proteins may also explain why we have not observed dysregulated *Tnf* expression in *Gpatch*^*−/−*^ animals. It is probable that *Tnf* expression in a given cell or tissue type is regulated by a particular complement of RBPs, as more than 20 AU-rich element-binding proteins have already been identified thus far [26], and over 1500 RBPs have been catalogued in human cells [27]. As such, a role for GPATCH2 in the regulation of *Tnf* expression at the level of the whole organism may be masked by functional overlap between two or several RBPs. A role for GPATCH2 in the control of *Tnf* expression may only become apparent when assessing specific cell types or when expression of other RBPs is also perturbed.

Of all tissues assayed, GPATCH2 protein expression was highest in the testis. However, *Gpatch*^*−/−*^ animals did not display breeding deficiencies and the morphology of the testis was normal. Thus, the significance of GPATCH2 expression in the testes remains to be identified. Our data also demonstrated expression of GPATCH2 in a variety of other tissues (albeit at lower levels compared to the testis), suggesting a role for this highly conserved protein beyond testis structure and function. *GPATCH2* mRNA was shown to be elevated in ~40% of samples in a gene expression analysis of 42 clinical breast cancer specimens [10]. There is also a suggestion that GPATCH2 can inhibit NF-κB activity in cell lines [9]. The function of GPATCH2 within the whole organism remains unclear as we did not identify any overt phenotype in our *Gpatch2*^*−/−*^ mice. While we were unable to determine a role for GPATCH2 in the control of *Tnf* expression, it would be interesting to assess the impact of the loss of GPATCH2 in the context of breast cancer and NF-κB activation, as this may uncover a role for GPATCH2 in vivo.

## Methods

### Immunoblotting

Organs were mechanically lysed with a TissueLyser II (Qiagen, Hilden, Germany) in RIPA buffer supplemented with cOmplete Protease Inhibitor Cocktail (Roche). BMDMs were lysed in RIPA buffer supplemented with cOmplete Protease Inhibitor Cocktail. Samples containing 40 µg total protein were reduced and denatured after addition of Laemmli buffer at 100 °C for 5 min, and separated on 4–12% Bis-Tris gels (Invitrogen) or 4–15% Criterion TGX stain free gels (BioRad) before blotting onto an 0.45 µm PVDF membrane (Merck). HRP-conjugated goat anti-rat or anti-mouse IgG secondary antibodies conjugated to horseradish peroxidase (HRP) (Southern Biotech), enhanced chemoluminescence (ECL) reagent (Immobilon) and a Chemidoc XRS + (Biorad, Hercules, California, USA) were used for detection and imaging of proteins. GPATCH2 specific antibodies from Boster Bio (A12468), Proteintech (24366-1-AP) and Biorbyt (orb157265) were tested. AC-74 monoclonal antibody (Sigma, A5316) was used for detection of β-actin. Uncropped immunoblots are shown in Supplementary Fig. 5.

### Recombinant GPATCH2 expression and monoclonal antibody generation

Exons 4–10 (amino acids 258–527) of mouse *Gpatch2* were cloned into T7 pET-15b plasmid (Novagen), and His-tagged recombinant GPATCH2 protein was affinity purified from BL21 *E. coli* (NEB) under native conditions using Ni-NTA resin (Invitrogen) and imidazole elution. Recombinant truncated GPATCH2 protein was used for immunisation of rats at the WEHI Antibody Facility, and B cell hybridoma supernatants were used at a dilution of 1/50 for detection of endogenous GPATCH2 in Fig. 2C. Hybridoma supernatant was concentrated 5-fold using Amicon 30K MWCO centrifugal filters (Millipore) then diluted 1/50 for GPATCH2 detection in Fig. 4F and Supplementary Fig. 1.

### Cell culture

To generate BMDMs, bone marrow was flushed from the mouse tibiae and femora and cultured in DMEM supplemented with 10% foetal calf serum (Sigma) and 20% L929-conditioned medium (a source of M-CSF) at 37 °C, 5% CO_2_ for 7 days. HEK293T cells were maintained at 37 °C, 10% CO_2_ in DMEM supplemented with 10% foetal calf serum. BMDMs were treated with 100 ng/mL *E. coli 0111. B4* LPS (Sigma). L929-conditioned medium was prepared from L929 cells cultured in DMEM (Gibco) +10% foetal calf serum.

### CRISPR screen and cell transfection

Cas9-expressing HEK293T cells were stably transfected with *GFP Tnf* 3’ UTR deletion reporter constructs (Supplementary Table 2) and selected with 100 µg/mL hygromycin (Thermo Fisher Scientific). *GFP Tnf* 3’ UTR deletion constructs contained the SV40 promoter sequence, GFP coding sequence and the mouse *Tnf* 3’ UTR sequence containing a deletion of either the ARE or the NRE [7]. To prepare lentiviral particles for stable transfection, HEK293T cells were co-transfected with pCMV-VSV-G (Addgene #8454), pCMV-dR8.2 (Addgene #8455), and the plasmid of interest using Fugene 6 (Promega). Single cells with moderate GFP expression were sorted by flow cytometry, and clonal populations stably transduced with the GeCKO v2 CRISPR sgRNA library [11] and selected with 2 µg/mL puromycin (Thermo Fisher Scientific). For each clonal population, cells with high GFP expression following GeCKO v2 library application were pooled and expanded. Genomic DNA was isolated from HEK293T cells with a DNeasy kit (Qiagen). Prior to sequencing, the GeCKO backbone vector which surrounds the guide RNA sequence was amplified with PCR primers containing the Illumina sequencing primer, bridge amplification sequences and vector-specific sequence. 76-base pair paired end reads were generated by next-generation sequencing (Illumina), and enriched sgRNAs in high GFP HEK293T cells mapped and quantified using Pipeline Pilot. Raw sgRNA counts are shown in Supplementary Table 1.

### Mice

*Gpatch2*^*−/−*^ mice were generated on a C57BL/6 background with CRISPR/Cas9 technology [28] using the protocol described in [16]. Exon 3 was deleted to engineer *Gpatch2*^*−/−*^ mice utilising two short guide RNAs. Genotyping of *Gpatch2* mutant mice was performed with a three-primer reaction. The sgRNA and genotyping primers are listed in Supplementary Table 3. F0 founders were backcrossed with wild-type C57BL/6 mice for two generations before generating *Gpatch*^*+/−*^ and *Gpatch2*^*−/−*^ animals as well as wild-type littermates for analysis. For LPS challenge, mice were injected intraperitoneally with *E. coli 0111.B4* LPS (Sigma) prepared in Dulbecco’s PBS and filter sterilised. Mouse behaviour and rectal temperature were observed every hour post-injection and mice needed to be sacrificed for ethical reasons when either i) their temperature fell below 33 °C or ii) behavioural scoring indicated clear discomfort. The personnel injecting, temperature monitoring and scoring LPS-injected mice were blinded to genotype. For SM-injection, mice were injected subcutaneously into their flanks with 100 µL 1 mg/mL Compound A (TetraLogic Pharmaceuticals) prepared in 12% Captisol (Cydex Pharmaceuticals). Automated blood analysis was performed using the Advia 2120 Blood Analyser (Siemens, Munich, Germany).

### Quantitative PCR

RNA was isolated from cells and tissues with TRIzol (Invitrogen) according to the manufacturer’s instructions and cDNA was synthesised with oligo(dT) primer and the Tetro cDNA Synthesis Kit (Bioline). The cDNA was amplified using SensiFAST SYBR Hi-ROX (Bioline) and analysed with a 384-well QuantStudio 12K Flex Real-Time PCR System (Thermo Fisher Scientific, Waltham, Massachusetts, USA). Oligonucleotide primers used for PCR are shown in Supplementary Table 3.

### ELISA

Blood obtained from cardiac or retro-orbital bleeds of mice was incubated at 37 °C for 10 min, chilled at 4 °C for 20 min, and centrifuged at 5000 × *g* for 2 min to separate serum. Skin of mice was mechanically lysed in protein extraction buffer (20 mM Tris pH 7.5, 150 mM NaCl, 1% Triton X-100, 2 mM EDTA, 10% glycerol) supplemented with cOmplete Protease Inhibitor Cocktail (Roche). Mouse TNF, IL-6 and MCP-1 were quantified with uncoated detection kits (Invitrogen) and read using the CLARIOstar microplate reader (BMG Labtech, Ortenberg, Germany).

### Flow cytometry

Cell sorting was performed using a BD FACSAria III (BD, Franklin Lakes, New Jersey, USA), and cells were analysed on a LSR II flow cytometer (BD). Fluorochrome conjugated antibodies against TCRβ, CD4, and CD8 were used to stain various T cell subsets. Fluorochrome conjugated antibodies against B220, IgM, IgD were used to identify different B cell subsets and fluorochrome conjugated antibodies against CD11b and GR-1 used to identify myeloid cell subsets. All antibodies used for flow cytometry were produced and conjugated to fluorochromes in-house and are listed in Supplementary Table 4.

### Statistical analysis

Significance in Kaplan–Meier survival curves were calculated using the log-rank test. *P* values from log-rank tests are shown, and a significant difference was defined as *p* ≤ 0.05. *P* values were otherwise calculated using the Mann–Whitney test, and only significant *p* values (*p* ≤ 0.05) are shown in the figures and supplementary figures.

## Supplementary information

Supplementary Materials Text

Supplementary Table 1

Supplementary Table 2

Supplementary Table 3

Supplementary Table 4

Supplementary Figure 1

Supplementary Figure 2

Supplementary Figure 3

Supplementary Figure 4

Supplementary Figure 5A

Supplementary Figure 5B

Supplementary Figure 5C

Supplementary Figure 5C continued

Reproducibility Checklist

## Data Availability

Uncropped Western blots are presented in Supplementary Fig. 5. All other primary data are available upon reasonable request.
