# Supplementary material for: Deletion of Gpatch2 does not alter Tnf expression in mice
Source: Cell Death Dis. 2023 Mar 27;14(3):214. doi: 10.1038/s41419-023-05751-x (PMC10043016; doi:10.1038/s41419-023-05751-x)
Supplement: Supplementary file 1 — Supplementary Materials Text [file 41419_2023_5751_MOESM1_ESM.docx]

**Destiny Dalseno et al: Supplementary Materials Text**

**Supplementary Figure 1. GPATCH2 is expressed in several mouse tissues.**

Our most sensitive hybridoma supernatant was concentrated 5-fold, and tissue lysates from wild-type (+/+) or *Gpatch2^-/-^* (-/-) mice probed for endogenous GPATCH2. The longer arrow indicates full-length mouse GPATCH2, and the shorter arrow a possible isoform of GPATCH2.

*(n=1)*

**Supplementary Figure 2. *Gpatch2^-/-^* histology.**

Representative H&E staining of the liver **(A)**, pancreas **(B)**, kidney **(C),** lung **(D),** spleen **(E),** and brain **(F)** from *Gpatch2^-/-^* mice. Scale bars: (A) - (D) 100 μm, (E) & (F) 200 μm.

**Supplementary Figure 3. Immune populations and solid lymphoid organ cell counts in wild-type and *Gpatch2^-/-^* mice.**

Flow cytometric analysis of immune cell composition in the bone marrow, spleen, lymph nodes, blood and thymus in wild-type and *Gpatch2^-/-^* mice. Total cell numbers (organ cell count) of the inguinal lymph nodes, spleen and thymus were also determined by flow cytometric analysis. Tissues were obtained from age- and sex-matched mice 6 - 14 weeks of age (females: n=3 per genotype, males: n=4 per genotype). White bars correspond to wild-type (WT), and grey bars to *Gpatch2^-/-^* throughout. Data are presented as mean ± SEM, each data point represents an individual mouse. Data from female mice n=1, data from male mice pooled from n=2 experiments. p ≤ 0.05 (*) defined as significant, only significant p values are shown (Mann-Whitney test).

**Supplementary Figure 4. Automated blood analysis of wild-type and *Gpatch2^-/-^* mice.** Cardiac bleeds were obtained from age- and sex-matched mice 6 - 14 weeks of age (females: n=3 per genotype, males: wild-type n=3, *Gpatch2^-/-^* n=4) and blood cell populations were determined by ADVIA. White bars correspond to wild-type (WT) mice and grey bars to *Gpatch2^-/-^* mice throughout. Data are presented as mean ± SEM, each data point represents an individual mouse. Data from female mice n=1, data from male mice pooled from n=2 experiments. No statistically significant p values were obtained from comparison of wild-type and *Gpatch2^-/-^* mice (Mann-Whitney test). WBC: white blood cells. RBC: red blood cells. PLT: platelets. HCT: haematocrit. RDW: RBC distribution width. HGB: haemoglobin. MCH: Main cell haemoglobin. CHCM: cellular haemoglobin concentration mean. HDW: haemoglobin distribution width.

**Supplementary Figure 5. Uncropped immunoblots.**

**(A)** Uncropped immunoblots relating to Figure 2C. **(B)** Uncropped immunoblots relating to Figure 4F. **(C)** Uncropped immunoblots relating to Supplementary Figure 1. Lines indicate lanes shown in manuscript.

**Supplementary Table 1. CRISPR screen results for HEK293T cells expressing the *GFP-Tnf Del* *NRE* reporter construct (unprocessed data, shown for each clone with hits from highest to lowest enriched sgRNA).**

**Supplementary Table 2. *Tnf* 3’ UTR deletion constructs.**

**Supplementary Table 3. Oligonucleotide primers used for PCR and qPCR.**

**Supplementary Table 4. Fluorochrome-conjugated antibodies for flow cytometry.**
