# Supplementary material for: Deletion of Gpatch2 does not alter Tnf expression in mice
Source: Cell Death Dis. 2023 Mar 27;14(3):214. doi: 10.1038/s41419-023-05751-x (PMC10043016; doi:10.1038/s41419-023-05751-x)
Supplement: Supplementary file 3 — Supplementary Table 2 [file 41419_2023_5751_MOESM3_ESM.docx]

| **Reporter Construct** | ***Tnf* 3’ UTR sequence deleted** |
| --- | --- |
| *GFP-Tnf Del ARE* | ctatttatatttgcacttattatttattatttatttattatttatttatttgcttatgaatgtatttatt |
| *GFP-Tnf Del NRE* | gctgatttggtgaccaggctgtcgctacatcactgaacctctgctccccacgggagccgtga  ctgtaatcgcccta |

**Supplementary Table 2. *Tnf* 3’ UTR deletion constructs.**
