# Supplementary material for: Deletion of Gpatch2 does not alter Tnf expression in mice
Source: Cell Death Dis. 2023 Mar 27;14(3):214. doi: 10.1038/s41419-023-05751-x (PMC10043016; doi:10.1038/s41419-023-05751-x)
Supplement: Supplementary file 4 — Supplementary Table 3 [file 41419_2023_5751_MOESM4_ESM.docx]

| **Primer** | **Sequence** |
| --- | --- |
| *Gpatch2* CRISPR sgRNA #1 | 5’- GGGAAAGTCGCACCCGTCGC |
| *Gpatch2* CRISPR sgRNA #2 | 5’- TATATGTGGACTTATCACGG |
| *Gpatch2* Genotyping Forward | 5’-TGGGAAATTCTCAGAGCAGC |
| *Gpatch2* Genotyping Reverse #1 | 5’-CAGATGCCGCCTTCTTCTTC |
| *Gpatch2* Genotyping Reverse #2 | 5’-GTGACTGCAGAGTGCTGACT |
| *Hprt* qPCR Forward | 5’-AGCAGTACAGCCCCAAAATG |
| *Hprt* qPCR Reverse | 5’-ATCCAACAAAGTCTGGCCTGT |
| *Tnf* qPCR Forward | 5’-ATCGGTCCCCAAAGGGATGAG |
| *Tnf* qPCR Reverse | 5’-TGCTCCTCCACTTGGTGGTTT |

**Supplementary Table 3. Oligonucleotide primers used for PCR and qPCR.**
