# Supplementary material for: Deletion of Gpatch2 does not alter Tnf expression in mice
Source: Cell Death Dis. 2023 Mar 27;14(3):214. doi: 10.1038/s41419-023-05751-x (PMC10043016; doi:10.1038/s41419-023-05751-x)
Supplement: Supplementary file 5 — Supplementary Table 4 [file 41419_2023_5751_MOESM5_ESM.docx]

| **Marker** | **Clone** |
| --- | --- |
| B220 | RA3-6B2 |
| CD4 | YTA3.2.1 |
| CD8 | 53.6.1.2 |
| CD11b | M1/70 |
| GR-1 | RB6-8C5 |
| IgD | 11-26C |
| IgM | 5.1 |
| TCRβ | H57-597.1 |

**Supplementary Table 4. Fluorochrome-conjugated antibodies for flow cytometry.**
