# Supplementary figures and images for: Deletion of Gpatch2 does not alter Tnf expression in mice
Source: Cell Death Dis. 2023 Mar 27;14(3):214. doi: 10.1038/s41419-023-05751-x (PMC10043016; doi:10.1038/s41419-023-05751-x)

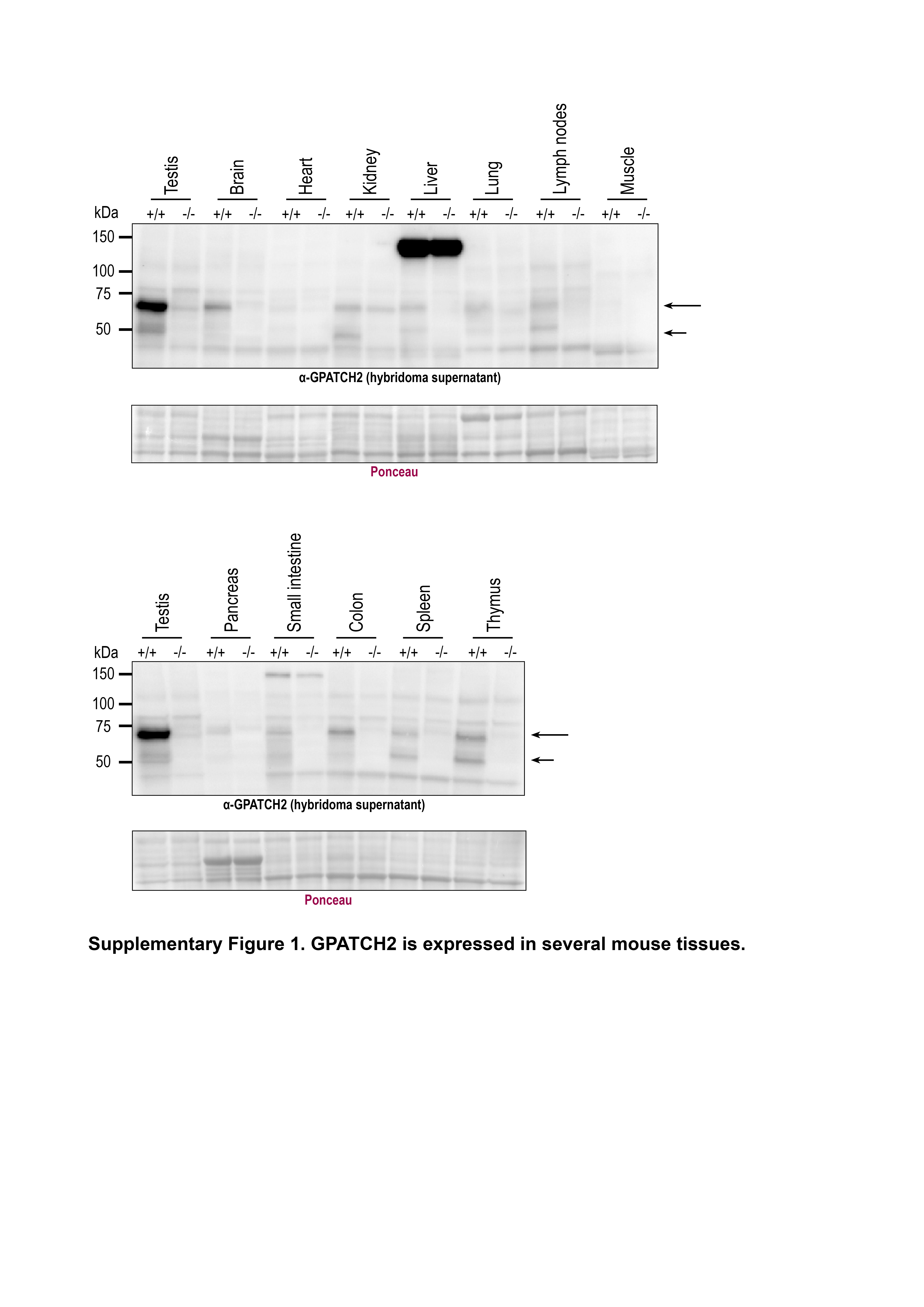

Supplement: Supplementary file 6 — Supplementary Figure 1 [file 41419_2023_5751_MOESM6_ESM.png]

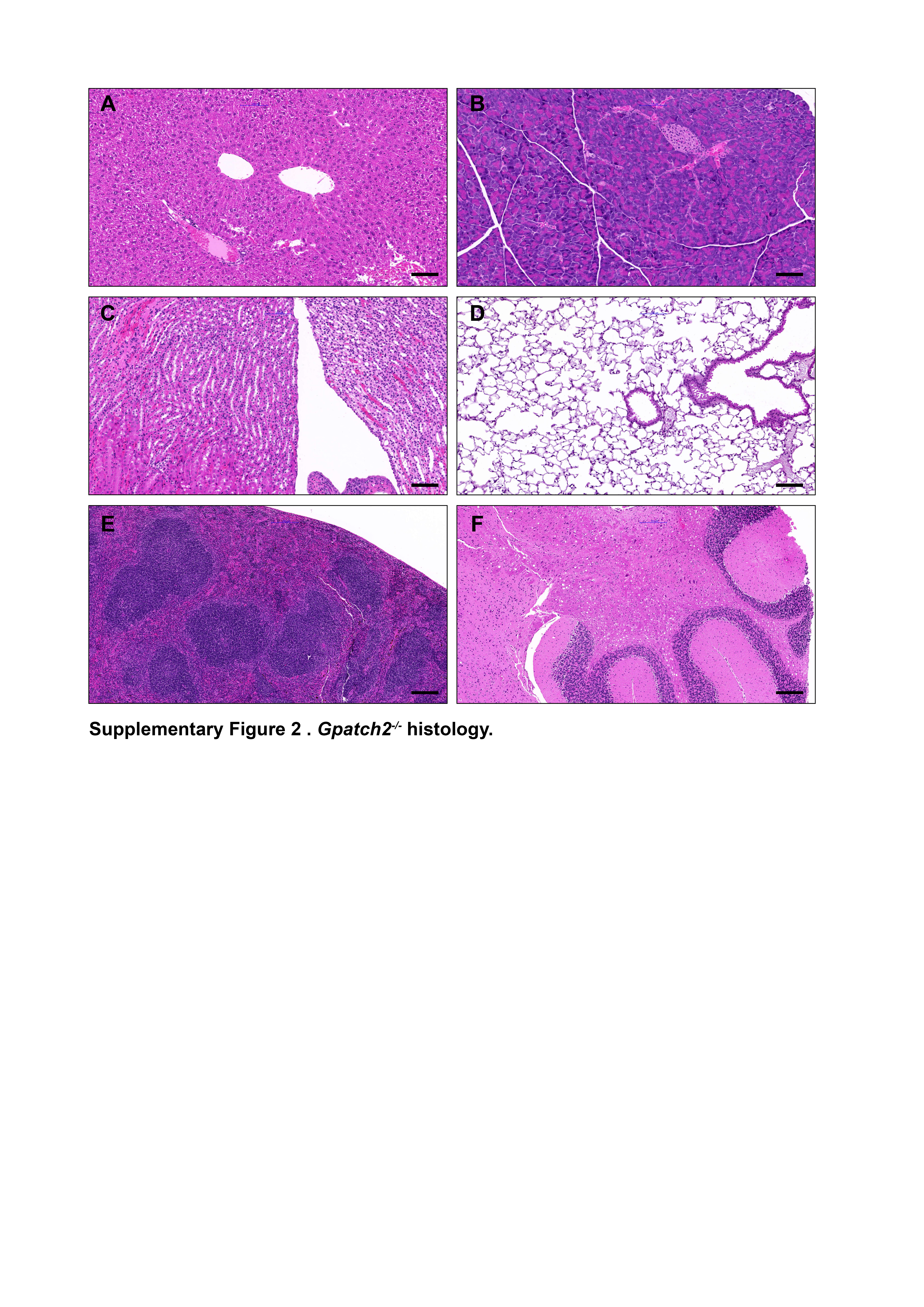

Supplement: Supplementary file 7 — Supplementary Figure 2 [file 41419_2023_5751_MOESM7_ESM.png]

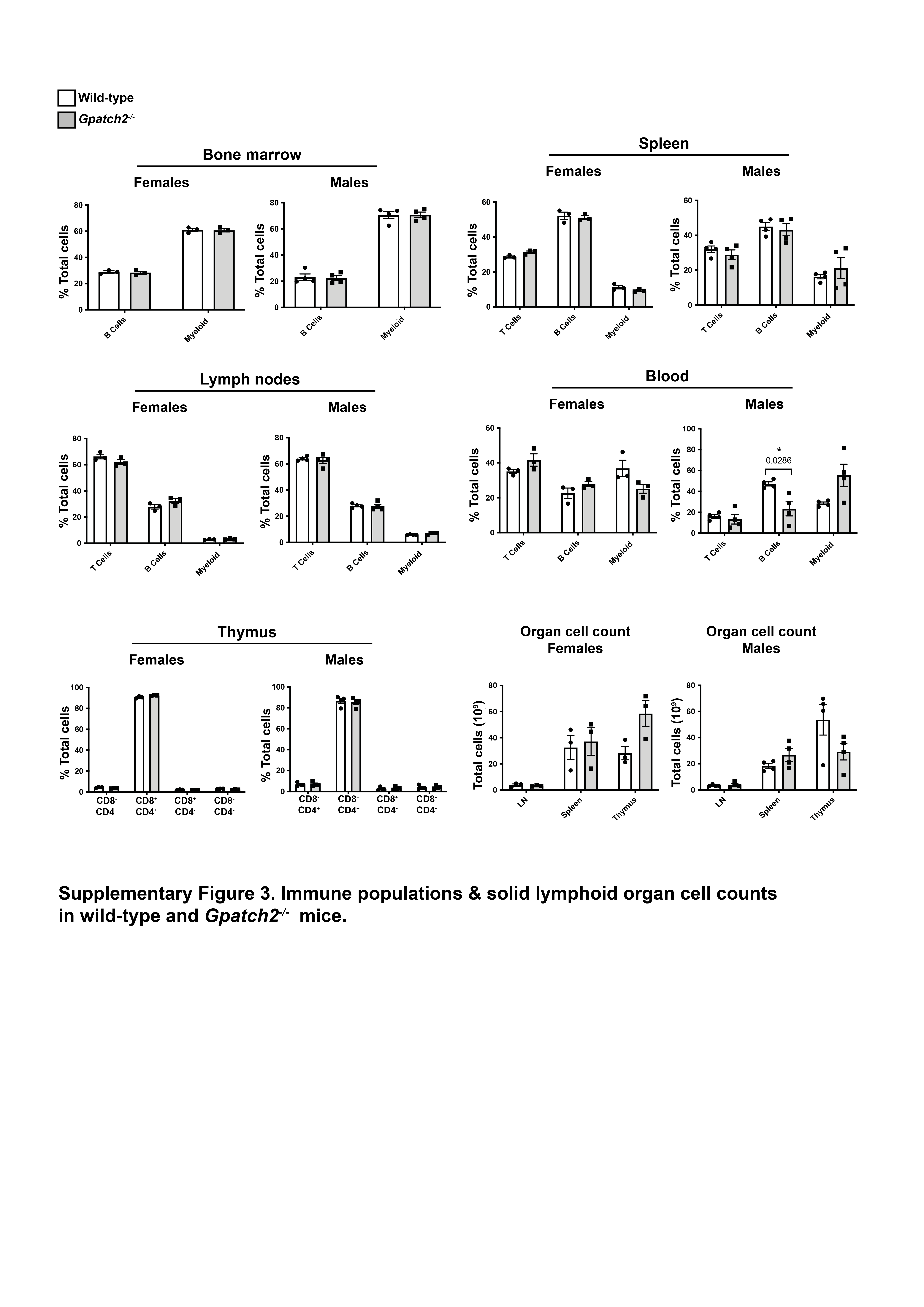

Supplement: Supplementary file 8 — Supplementary Figure 3 [file 41419_2023_5751_MOESM8_ESM.png]

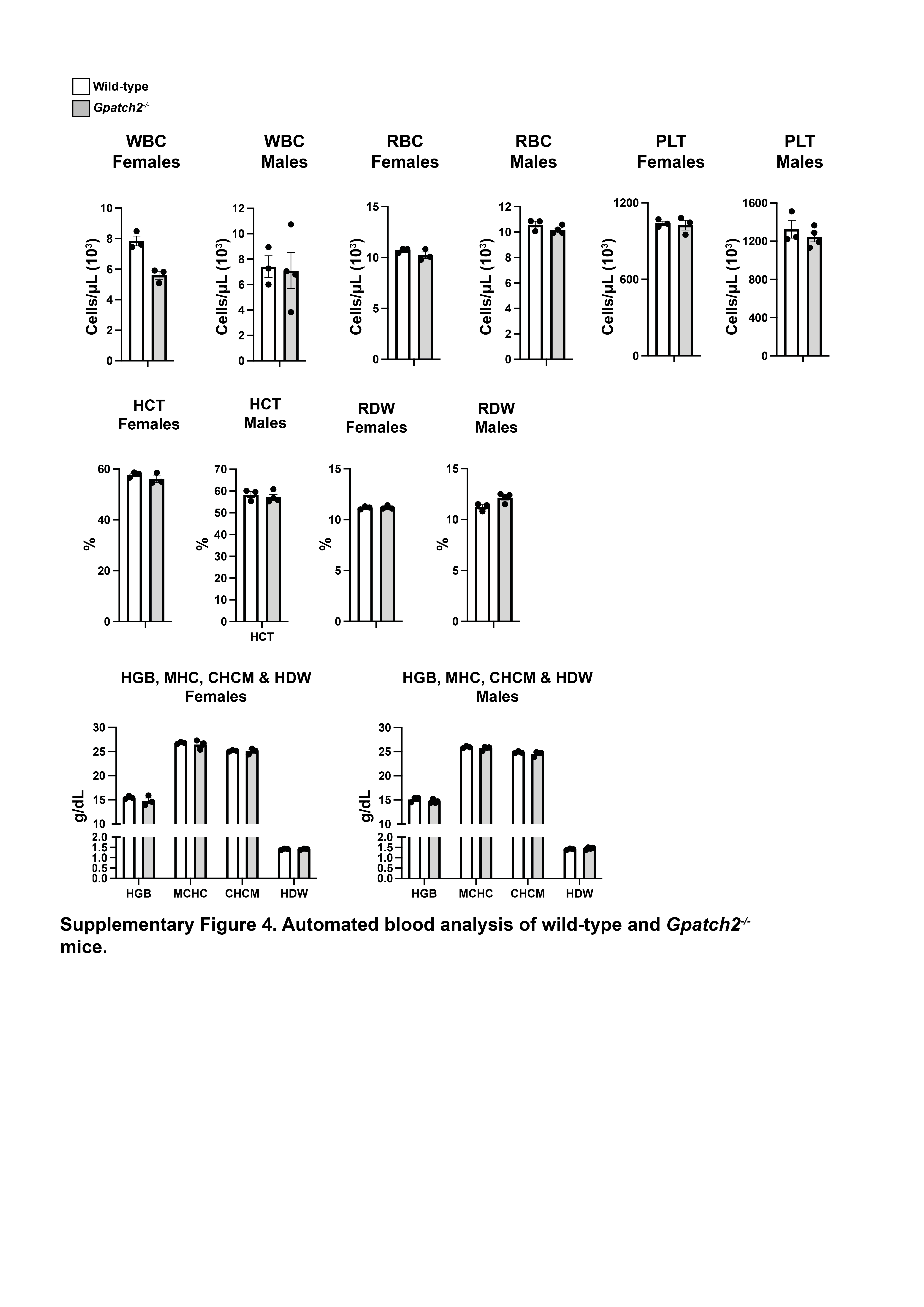

Supplement: Supplementary file 9 — Supplementary Figure 4 [file 41419_2023_5751_MOESM9_ESM.png]

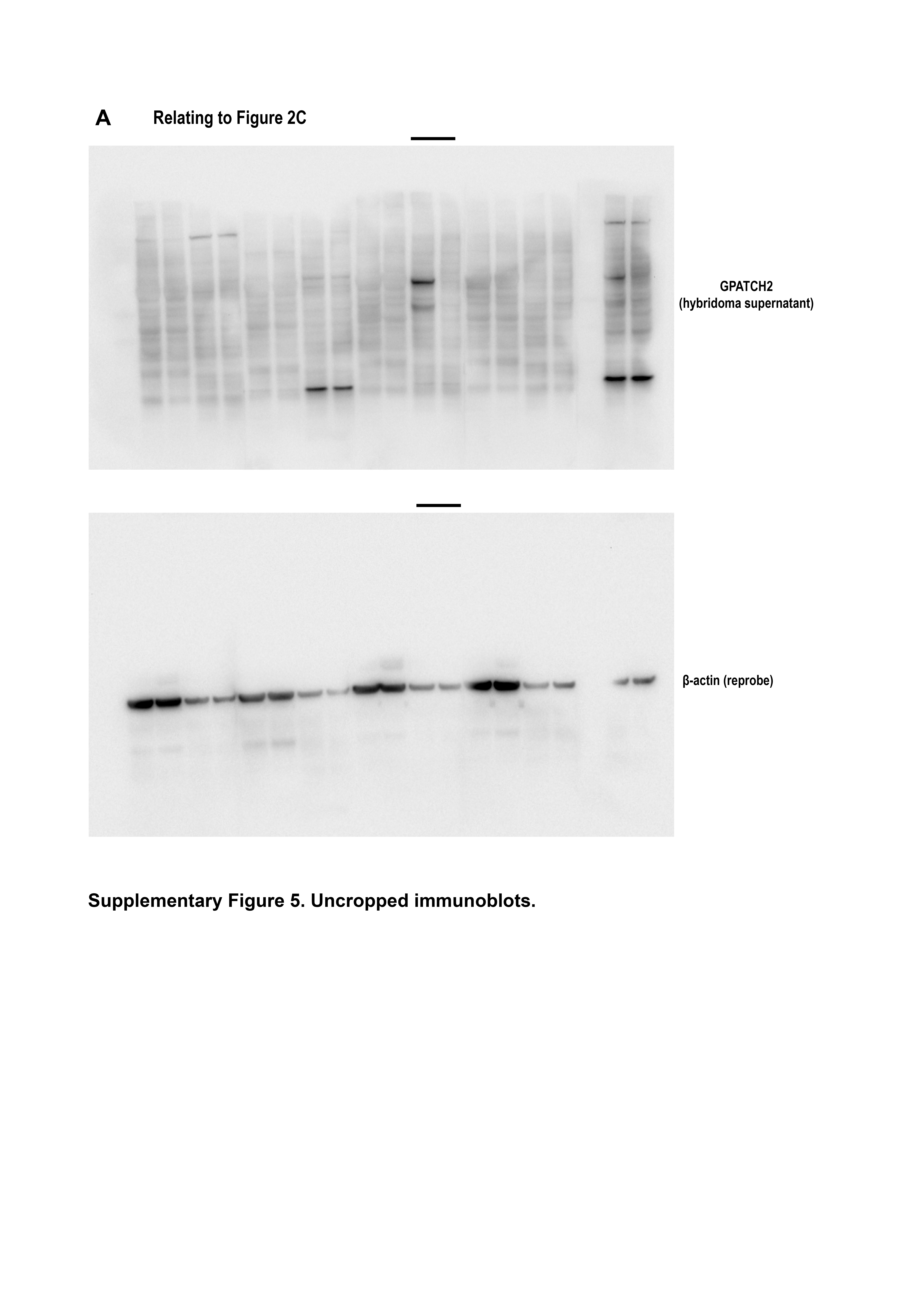

Supplement: Supplementary file 10 — Supplementary Figure 5A [file 41419_2023_5751_MOESM10_ESM.png]

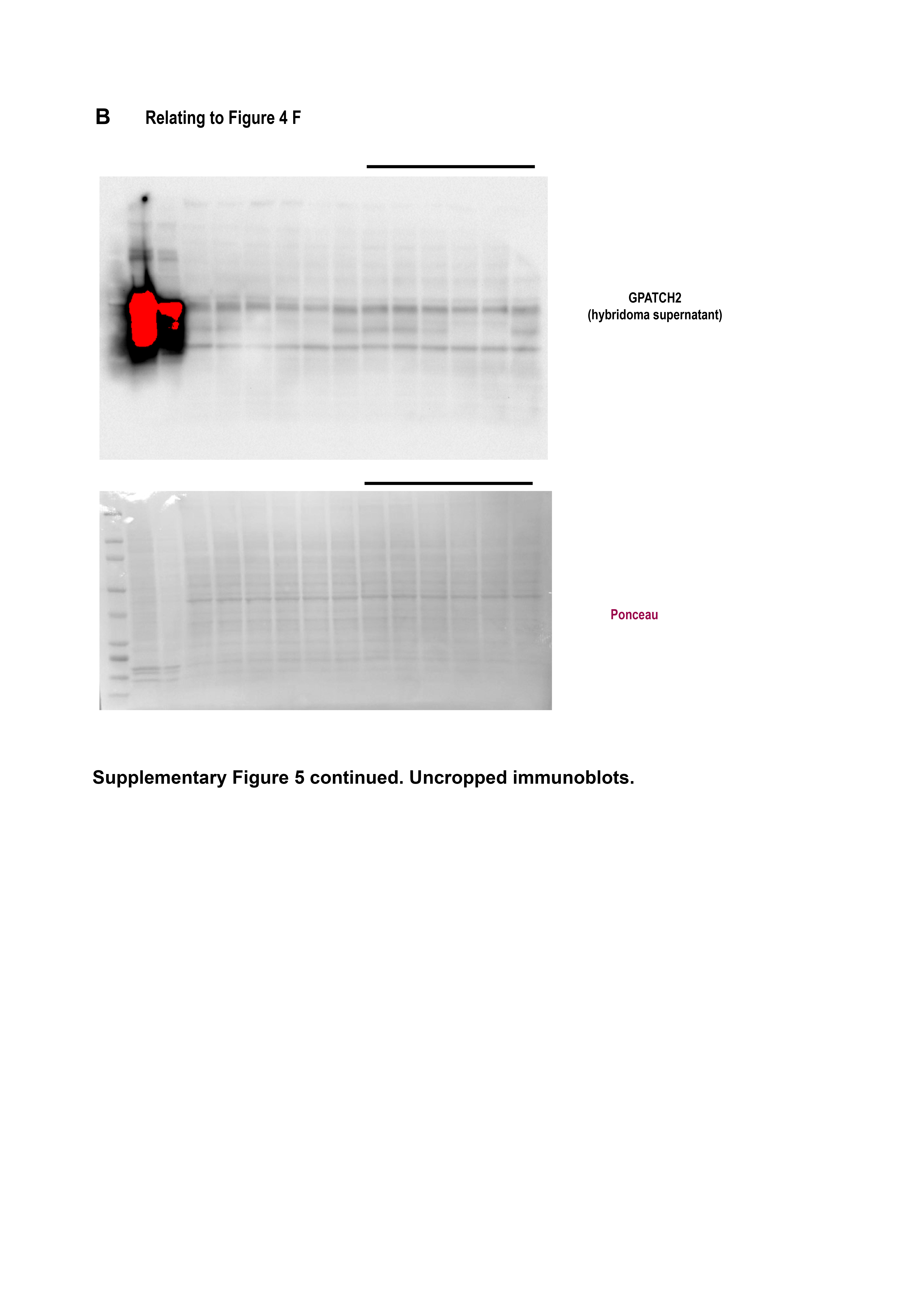

Supplement: Supplementary file 11 — Supplementary Figure 5B [file 41419_2023_5751_MOESM11_ESM.png]

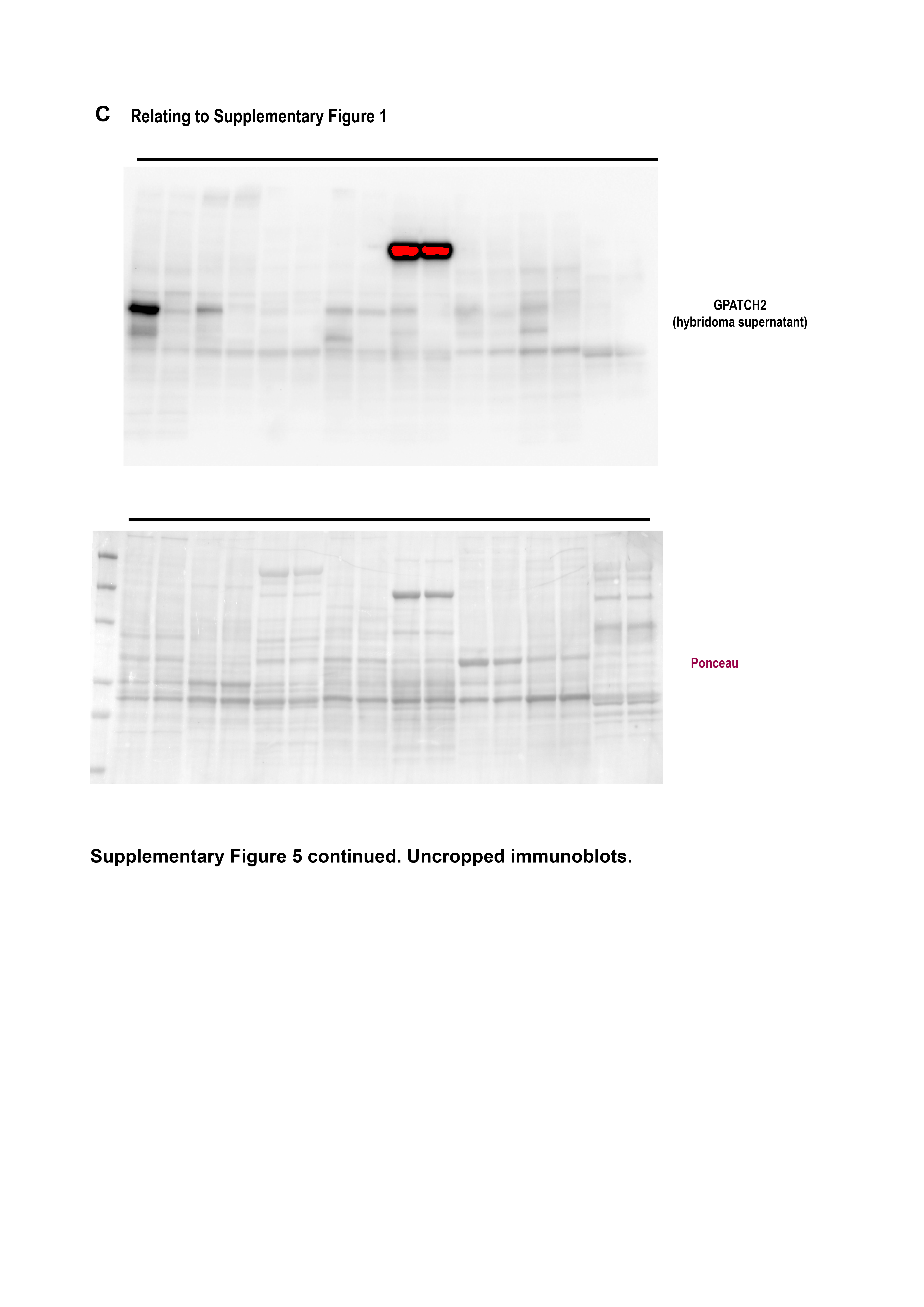

Supplement: Supplementary file 12 — Supplementary Figure 5C [file 41419_2023_5751_MOESM12_ESM.png]

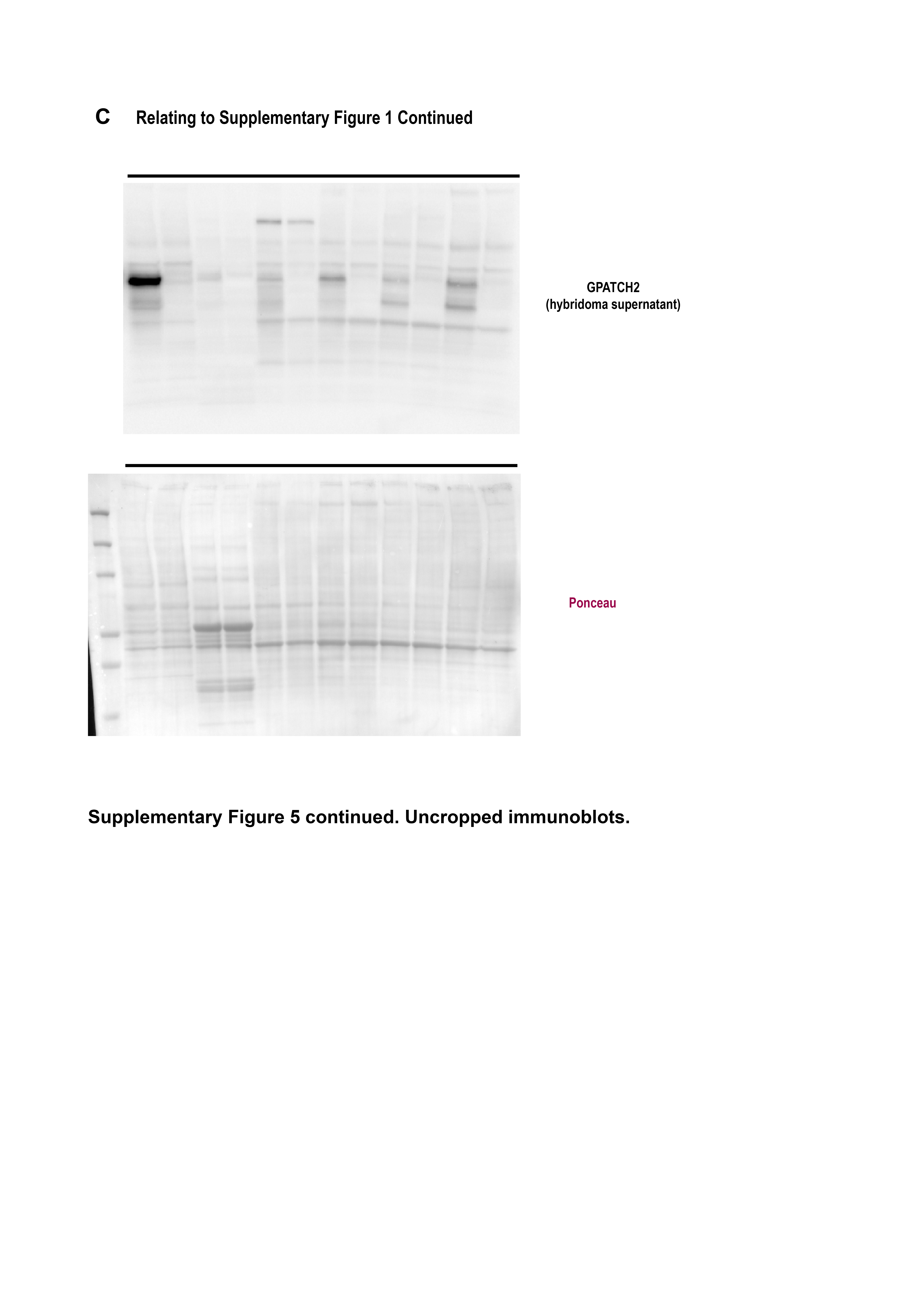

Supplement: Supplementary file 13 — Supplementary Figure 5C continued [file 41419_2023_5751_MOESM13_ESM.png]
